# Connections of bullying experienced by *Kyokushin karate* athletes with the psychological state: is “a Cure for Bullying” safe?

**DOI:** 10.3389/fspor.2024.1304285

**Published:** 2024-01-25

**Authors:** Jolita Vveinhardt, Magdalena Kaspare

**Affiliations:** Institute of Sport Science and Innovations, Lithuanian Sports University, Kaunas, Lithuania

**Keywords:** *Kyokushin karate*, bullying in sport, psychological state, qualification, martial arts, athletes, adults

## Abstract

Although some authors propose practicing martial arts as a prevention against bullying, little is known about bullying among karate athletes and the consequences of negative behaviour for athletes' psychological state. This study aims to explore the effect of bullying on the psychological state of *Kyokushin karat*e athletes. A total of 371 athletes were surveyed to measure bullying experiences and signs of stress, anxiety, and depression. It was determined that 75.5% of *Kyokushin karate* athletes had experienced unethical behaviour by their coaches or other athletes towards them at least once, and the scores on the signs of stress, anxiety, and depression correlated with damage in the fields of communication, social relations, and physical health. The results of binary logistic regression have shown that the qualifications of karate athletes and their participation in competitions influence the risk of being bullied. Organisations in which *Kyokushin karate* athletes do sports should devote more attention to preventing bullying among karate athletes themselves, especially when preparing for competitions as bullying can harm communication, social relations, and physical health.

## Introduction

The sport *of karate* is a widely practised form of self-defence and is considered a discipline that facilitates physical and mental balance and improving health (Greco et al.) (1, 2). A study conducted by Oulanova (3) showed that martial arts could help people regulate their behaviour and improve their mental health by learning self-control and concentration. Therefore, it is believed that martial arts can also be an effective tool in the fight against bullying and its consequences for athletes' health Greco et al. 2019 (1, 2, 4, 5). Lafuente et al. (6) maintain that training in traditional martial arts can be an effective means to reduce aggression and anger.

However, the branches of combat sports differ in their level of aggressiveness. A study by Piepiora et al. (7) showed that the semi-contact (*Shotokan karate*) system distinguished itself by a significantly lower overall level of aggressiveness compared with other *Kumite* systems (when athletes performed offensive and defensive actions with their hands and feet). Meanwhile, according to the author, *Kyokushin* karate is characterised by a significantly higher overall level of aggressiveness in physical, verbal, and social aspects. Bullying is considered a form of aggressive behaviour (8), and in the sports environment, where competition and aggressive behaviour are considered a norm, victimisation may be treated as a normal expression of aggression but not bullying (9, 10). For this reason, some acts of bullying may remain unrecognised. Furthermore, the motives behind the choice of karate are also important. It has been noticed that attending martial arts classes may be associated with a wish to defend oneself against bullying (11). However, a significant proportion of former victims of bullying later become persecutors themselves (12). In other words, karate may not be immune to bullying and may simply go unnoticed.

Other important aspects are related to the benefits of karate to the athlete well-being. Although Greco et al. (1, 2) linked the preventive effect of karate with self-regulation, fostered respect, and health promotion, the study conducted by Pinto-Escalona et al. (13) showed only a positive effect on health and academic achievement. Compared to the control group, engaging in karate had no significant impact on variables such as psychosocial difficulties, emotional symptoms, or prosocial behaviour. Therefore, the results are inconclusive. In another study that was based on a meta-analysis of 12 studies, Harwood et al. (14) noted that aggression (anger, violence) related to problem behaviour among young athletes practising different martial arts styles decreased, but the authors did not rule out the possibility that age could still be a potential moderator. Furthermore, the positive effect may also depend on the duration of training (14). Research in this area is still scarce and is often only indirectly related to bullying or conducted among school-age karate practitioners.

Thus, the prevalence of bullying among karate athletes of different age groups remains unknown. Hence, addressing the topic of karate as a bullying prevention mechanism leaves important unknowns related to the dynamics of bullying within the sport of karate itself. Therefore, this study aims to explore the effect of bullying on the psychological state of *Kyokushin karate* athletes.

This is an exploratory study. Specifically, it explores how actions harming interpersonal communication (15, 16), undermine social context within the athlete group and damage personal health, unfavorable impact psychologically, emotionally, socially (17) and performance areas are related to the scores of athlete stress (18, 19), anxiety (20, 21), and depression signs (22). Damage to communication (23) in this study includes hostile verbal communication by insulting, shouting, or limiting the target's opportunities for self-expression. Social undermining manifests itself by demonstrative actions that damage the athlete's reputation by spreading gossip, underestimating him or her as a person and as an athlete (15, 16). Damage to health and performance (16, 24) encompasses physical (25) and material damage (26), actions that disrupt the athlete's performance. It is also sought to find out the prevalence of these negative experiences and how likely athletes are to experience bullying, depending on their age Stirling et al. (27) and the nationality of the athlete (28), gender, sports qualification and duration of sporting activities, also preventive strategies (29). This study contributes to a small but growing body of literature aimed at understanding the manifestation of bullying among *Kyokushin karate* athletes in order to assist those who are responsible for a safe and healthy sporting environment.

## Literature review

### Bullying in sport

Bullying is defined as the repeated hostile behaviour of one or several individuals against another person with the intent to harm, both physically and emotionally, and includes any resulting damage (24). Bullying increases the level of stress (18, 19) and negatively affects athletes' involvement in sports and their well-being (30). In addition, being a victim of bullying is associated with a higher susceptibility to various diseases (18) and greater risk of depression (22).

Stirling et al. (27) note that bullying is experienced not only by young people who do sports but also by adults, as the phenomenon is based on power imbalance that is not related to the athlete's age. Areas of damage to athletes include damage to communication (negative verbal and non-verbal communication), social undermining (undermining the person's reputation), damage to health and performance (16).

The causes of bullying in sports are related to the characteristics of the sports environment and athletes’ individual traits (19, 30, 31). Muhsen and Mohsin (32) link bullying to sports culture, where aggressive behaviour is often perceived as “natural”, while this problem is not addressed at the levels of athletes, coaches and authorities. In addition to contextual environmental factors, the personality traits of bullies also play a role. The study by Sentse et al. (33) showed that bullying behaviour among school-age children was related not only to the desire to gain but also to maintain dominance and status in the group. Other studies highlighted aggressors' Machiavellianism (34) and oppression, anxiousness, and low self-esteem of the targets of bullying (35). However, there are no clear answers to what extent bullying causes depression or whether the depressive effect is a risk factor for subsequent bullying (36). In comparison, neuroticism in adult professional settings can predict both victimisation and the consequences of bullying (37).

### Kyokushin karate

It is a fighting style developed by M. Oyama in Japan in the middle of the last century. “*Karate Kyokushin* is characterized by competition in the knockdown formula. The fight takes place in close contact and allows blows to be inflicted with full force. The fight takes place without any protection (except for the suspension and the protector; for women, the chest protector)” [(38) p. 36]. Athletes' qualification is evaluated according to the rank system from 10 (orange belt, the lowest) to 1 (brown belt with black stripe) Kyu and from 1 (black belt with one gold stripe) to 10 Dan (black belt with ten gold stripes, the highest). It differs from other martial arts by its peculiar philosophy. M. Oyama, the founder of the *Kyokushin* karate style, promoted his own *Kyokushin* philosophy or the ultimate truth, which he derived from various Far Eastern schools of thought and identified with the Warrior's Way (39). As stated by Zabjek (40), it is believed that daily practice provides meaning to life, a sense of belonging, mental well-being, and spiritual support and, at the same time, also gives guidance on values, etiquette, and interpersonal relations. In this context, shared values enable developing trust-based interpersonal relationships that encourage seeking and accepting spiritual support (41, 42). In addition, close-contact fighting and difficult challenges are associated with real power, which in addition to the ability to defeat the opponent includes overcoming one's own weakness, pain, and fear (43).

Some differences are found when comparing different styles and individual qualities of *Kyokushin karate* athletes. Compared to kickboxing, no significant personality differences among *Kyokushin* athletes were observed: low neuroticism, high extraversion, conscientiousness, and moderate openness to experience were identified. Several studies have demonstrated that the level of qualification of athletes is related to a better psychological state. It has been found that the use of mindfulness practice and higher qualification in sport led to better psycho-emotional and achievement indicators (20). Another study found that masters of sports were characterised by a higher level of openness to experience than younger athletes, which is related to experience and the influence of pro-health values of karate (38). However, compared with aikido, the highest negative indicators of personality traits according to social norms (aggression-hostility) came from the *Kyokushin* group (44). Although genetic and social environmental factors may play a role, Litwiniuk et al. (44) state that the degree of physical contact in sports competitions is directly proportional to aggressiveness. Although aggressiveness in sport is important, and aggression does not necessarily turn into bullying, research shows that increased aggression is related to higher levels of bullying (9, 45).

### Martial arts and bullying

Because karate emphasizes respect, self-regulation, and health promotion, Greco et al. (1, 2) state that this martial art increases resistance to bullying. The results of the study conducted by these authors showed that after a 12-week intervention in which pupils aged 14–16 years trained in Shotokan karate, indicators of general resilience and well-being were higher than in the control group. The aforementioned authors maintain that this can reduce the likelihood that young people will engage in aggressive behaviour or experience bullying. Increased resilience among school-age pupils, like self-efficacy, was also identified after martial arts-based psychosocial intervention, which included psychoeducation (on bullying, self-respect, courage, goal-setting, values, and other topics), physical exercises, breathing, meditation, and combinations of blocks and strikes (46). The latter study, unlike the study by Greco et al. (1, 2), analysed the themes of peer pressure and bullying.

However, there are studies showing that bullying also exists among martial arts practitioners. This is demonstrated in an ethnographic study conducted in the martial arts school in Dengfeng, China. Dong (47), the author of this year-long study, observed that by practising martial arts, “the ‘bullies’ who were marginalised in academic schools” became leaders who used violence against the weaker. In addition, Orak et al. (48) in Turkey found that combat sports representatives had a strong inclination to direct their negative emotions towards those who were weaker, to demonstrate attitudes in order to justify their bullying behaviour, to indulge in bullying and establish superiority based on individual strength.

## Material and methods

### Instruments

The scales identifying bullying in sport (Damage to communication, Social undermining and Damage to health and performance) were developed based on previous research on workplace mobbing (15) and bullying in sport (16), summarising the negative behaviours described in those studies in the verbal, social, and physical domains (49). The items developed for the study to measure the signs of bullying in a sports context showed promising psychometric characteristics, and a validation article is forthcoming. Bullying experiences were measured using a 7-point scale: “Almost always (everyday)”, “Very often (every second/third day)”, “Quite often (once a week)”, “Not often (once in two weeks)”, “Very rarely (once in a month)”, “Almost never (once in 3–6 months)”, and “Never”. The participants were dichotomized between those who had answered “Almost never” to at least one item per dimension of bullying as having had an experience of bullying.

The Depression, Anxiety, and Stress Scale is a set of three self-report scales designed to measure the psychological states of depression, anxiety, and stress (50). Every scale consists of seven items. For example, items such as “I was unable to become enthusiastic about anything” (depression signs, further, DS, seven items), “I found it difficult to relax” (stress signs, further, SS, seven items), and “I was worried about situations in which I might panic and make a fool of myself” (anxiety signs, further, AS, seven items).

Questions measuring the characteristics of athletes [duration of participation in sports, athlete qualification (*Dan* and *Kyu*), membership in the national team, participation in competitions, meditation, training], and demographic characteristics (gender, age) were also integrated.

Questions measuring the characteristics of athletes [duration of participation in sports, athlete qualification (*Dan* and *Kyu*), membership in the national team, participation in competitions, meditation, training], and demographic characteristics (gender, age) were also integrated.

### Participants and procedures

Any adult engaged in practicing Kyokushin karate in Lithuania was eligible to participate in the study. Athletes were contacted by email containing information about the study. The purpose, guarantees of anonymity, confidentiality, the rights of the respondents were explained. This survey involved *N* = 371 *Kyokushin karate* athletes, of whom 59.3% were male and 40.7% were female. The respondents' age fluctuated from the late twenties to mid-thirties (*M* = 27.75 years, SD = 8.56, Min.—18, Max.—56). Most participants had done *Kyokushin* karate for at least 11 years (53.6% of all participants), 14.8% had done the sport for up to 5 years, and 31.5% had done it for 6–10 years. The more experience in karate that the athletes had, the higher the belts they also have [*χ*² (4) = 240.9, *p* < 0.001]; 71.4% of *Kyokushin* karate athletes who have been engaged in this sport for at least 11 years have the 1st Dan or a higher belt.

The study was conducted using an electronic questionnaire, the link of which was sent to the athletes who gave their consent to take part in the study. The questionnaire was protected against re-completion, and incomplete questionnaires were not accepted.

While analyzing the collected data, percentages of experienced bullying among athletes were calculated, and differences between groups based on the duration of karate practice, possession of a karate belt, participation in competitions, gender, and other factors were examined using Chi-square (*χ*^2^) tests. Additionally, correlations between bullying and indicators of stress, anxiety, and depression were computed. To determine the dependency of bullying on binary variables (experienced bullying or not), binary logistic regression was applied, considering variables such as age, gender, karate belt level, duration of sports practice, frequency of participation in competitions, and other relevant factors.

## Results

It has been found that 42.6% of karate athletes were harmed through communication (not allowed to express an opinion—CO1, spoken to in a raised tone—CO2, insulted, called swear words—CO3, had no dialogue with the coach—CO4). There is no significant difference between those who have experienced bullying in communication and the duration of practising karate, so experiencing bullying does not depend on experience. Owning a karate belt, participating in competitions, belonging to the national team, and coaching children or adults (training in groups of children or adults) do not differ significantly between athletes who have experienced harm through communication and those who have not experienced bullying. A difference in the experience of harm through communication was identified based on gender *χ*^2^ = 8.564, *p* = 0.003. The average age of athletes who experienced bullying (damage through communication) was 27.3 ± 8.2 years, and the oldest respondent who reported bullying in the field of communication was 53 years old (Table 1).

**Table 1:** Harm via communication: distribution by subgroups of characteristics of athletes in sport and demographic characteristics.

Factors	Subgroups	Damage to communication				*χ*²	*p*	*r*
		Inexperienced		Experienced				
		Cases	Percent	Cases	Percent			
Engaging in karate	Up to 5 years	31	14.6%	24	15.2%	0.037	0.982	−0.009
	6–10 years	67	31.5%	50	31.6%			
	11 years and more	115	54.0%	84	53.2%			
Karate belt	10-7 Kyu	19	8.9%	9	5.7%	2.281	0.320	−0.012
	6-1 Kyu	94	44.1%	80	50.6%			
	1 Dan and higher	100	46.9%	69	43.7%			
Participated in karate competitions	Yes	192	90.1%	148	93.7%	1.476	0.224	−0.063
	No	21	9.9%	10	6.3%			
Member of the national team	Yes	103	48.4%	77	48.7%	1.014	0.314	−0.052
	No	110	51.6%	81	51.3%			
Train children or adults	Yes	78	36.6%	66	41.8%	0.564	0.453	0.039
	No	135	63.4%	92	48.2%			
Gender	Male	140	63.6%	80	50.6%	8.564	0.003	0.152 *p* = 0.003
	Female	73	34.3%	78	49.4%			
Meditation effect	No	32	15.0%	26	16.5%	0.141	0.707	0.015
	Yes	181	85.0%	132	83.5%			
Frequency of participation in competitions	1–2 times	61	28.6%	43	27.7%	0.502	0.919	0.020
	3–4 times	63	29.6%	46	29.1%			
	5 times and more	39	18.3%	27	17.1%			
	Not participating	50	23.5%	42	26.6%			
Total		213	100%	158	100%			

Scores of stress, anxiety, and depression signs weakly but correlated with damage experienced by respondents due to bullying in communication (stress *r* = 0.274, anxiety *r* = 0.242, depression signs *r* = 0.225, *p* < 0.001). Thus, as bullying in communication increases, so do the signs of stress, anxiety, and depression experienced by *Kyokushin karate* athletes.

A large minority (37.2%) of karate athletes stated that they had experienced bullying, harm to their social relations (avoided contact—SU1, are viewed as an “empty place”—SU2, are mocked at, ridiculed—SU3, gossip was spread—SU4, decisions/proposals are publicly questioned—SU5, achievements in sports are undermined—SU6).

The duration of engaging in karate, possessing a karate belt, belonging to the national team, coaching others, gender, practising meditation, and feeling the benefits of karate do not have a significant difference in relation to experienced bullying when social relations are harmed. Bullying is equally characteristic regardless of the possessed karate belt or duration of engaging in sport. However, a significant very weak association/correlation was found between participation in competitions and bullying by applying isolation actions (*r* = −0.105, *p* = 0.042); i.e., if the athlete does not participate in competitions, he or she is less likely to experience bullying when the attacks are performed through social relations (Table 2).

**Table 2:** Social undermining: distribution by subgroups of characteristics of athletes in sport and demographic characteristics.

Factors	Subgroups	Social undermining				*χ*²	*p*	*r*
		Inexperienced		Experienced				
		Cases	Percent	Cases	Percent			
Engaging in karate	Up to 5 years	37	15.9%	18	13.0%	0.563	0.755	0.034
	6–10 years	73	31.3%	44	31.9%			
	11 years and more	123	52.8%	76	55.1%			
Karate belt	10-7 Kyu	22	9.4%	6	4.3%	3.66	0.161	0.077
	6-1 Kyu	110	47.2%	64	46.6%			
	1 Dan and higher	101	43.3%	68	49.3%			
Participated in karate competitions	Yes	209	89.7%	131	94.9%	3.093	0.079	−0.091
	No	24	10.3%	7	5.1%			
Member of the national team	Yes	113	48.5%	67	48.6%	0.00	0.92	−0.001
	No	120	51.5%	71	51.4%			
Train children or adults	Yes	82	35.2%	62	44.9%	3.458	0.063	−0.097
	No	151	64.8%	79	55.1%			
Gender	Male	141	60.5%	79	57.2%	0.384	0.536	0.032
	Female	92	39.5%	59	42.8%			
Meditation effect	No	36	15.5%	22	15.9%	0.016	0.900	0.007
	Yes	197	84.5%	116	84.1%			
Frequency of participation in competitions	1–2 times	61	26.2%	43	31.2%	5.516	0.013	−0.105 *p* = 0.042
	3–4 times	64	27.5%	45	32.6%			
	5 times and more	41	17.6%	25	18.1%			
	Not participating	67	28.8%	25	18.1%			
Total		233	100%	138	100%			

The average age of persons who experienced social isolation was 26.26 ± 7.73 SD years. The youngest person who experienced bullying through the acts of isolation was 18 years old and the oldest was 54 years old. Damage to social relations correlates with respondents' experienced signs of stress, anxiety, and depression (stress *r* = 0.177, *p* = 0.001, anxiety *r* = 0.160, *p* = 0.001, depression signs *r* = 0.155, *p* = 0.003).

Seventeen percent of karate athletes stated that they had experienced at least one type of harm to their health and performance (physical violence—HP1, assigned too easy tasks regardless of physical capabilities—HP2, financial penalty—HP3, material expenses—HP4).

The average age of athletes who stated that their health and results had been harmed was 27.23 ± 7.5 years. Bullying through being assigned tasks that are too easy differs depending on gender (*p* = 0.045). When analysing factors according to damage to athletes’ health and performance between different factors, no significant relationship was found in any of the subgroups. Thus, regardless of the duration of engaging in sport, possession of a karate belt, engaging in a meditation group, frequency of participation in competitions, and gender, bullying through harm to health and performance is experienced by athletes in a similar way (Table 3).

**Table 3:** Harm to health and performance: distribution by subgroups of characteristics of athletes in sport and demographic characteristics.

Factors	Subgroups	Damage to health and performance				*χ*²	*p*	*r*
		Inexperienced		Experienced				
		Cases	Percent	Cases	Percent			
Engaging in karate	Up to 5 years	45	14.5%	10	15.9%	0.248	0.883	−0.024
	6–10 years	96	31.2%	21	33.3%			
	11 years and more	167	54.2%	32	50.8%			
Karate belt	10-7 Kyu	24	7.8%	4	6.3%	0.511	0.775	−0.016
	6-1 Kyu	142	46.1%	32	50.8%			
	1 Dan and higher	142	46.1%	27	42.9%			
Participated in karate competitions	Yes	282	91.6%	58	92.1%	0.017	0.895	−0.007
	No	26	8.4%	5	7.9%			
Member of the national team	Yes	152	49.4%	28	44.4%	0.193	0.461	−0.023
	No	156	50.6%	35	55.6%			
Train children or adults	Yes	118	38.3%	26	41.3%	0.271	0.602	0.027
	No	190	61.7%	37	58.7%			
Gender	Male	180	58.4%	40	63.5%	0.553	0.457	−0.039
	Female	128	41.6%	23	36.5%			
Meditation effect	No	48	15.6%	10	15.9%	0.003	0.954	0.003
	Yes	260	84.4%	53	84.1%			
Frequency of participation in competitions	1−2 times	85	27.5%	19	30.2%	0.174	0.982	−0.018
	3–4 times	91	29.5%	18	28.6%			
	5 times and more	55	17.9%	11	17.5%			
	Not participating	77	25.0%	15	23.8%			
Total		308	100%	63	100%			

Bullying by harming health and performance correlates, although weakly, with experienced stress, anxiety, and depression (stress *r* = 0.241, *p* < 0.001, anxiety *r* = 0.238, *p* < 0.001, depression *r* = 0.225, *p* < 0.001). Scores indicating a poorer psychological state were higher among *Kyokushin karate* athletes who were negatively affected through health and achievements.

In addition, logistic regression (Table 4) was performed with respect to two groups (athletes who experienced bullying and athletes who did not experience bullying) and factors such as gender, age, participation in competitions, and possession of a karate belt. The results of the binary logistic regression show that the sensitivity of the model in predicting athletes who experienced bullying is 58 percent HL (the Hosmer and Lemeshow test) *χ*²=3.108, df = 8, *p* = 0.927, which shows that the model is an adequate fit. Pseudo-R-squared coefficient of determination (*R*^2^) – 0.049.

**Table 4:** Logistic regression with respect to athletes who experienced bullying vs. athletes who did not experience bullying.

Subgroups	*B*	SE	*p*	OR	PI 95% OR
Engaging in karate sport					
Up to 5 years^a^			0.431		
6–10 years	0.485	0.449	0.280	1.625	0.674–3.919
11 years and more	0.284	0.274	0.300	1.329	0.776–2.275
Karate belt					
10-7 Kyu^a^			0.045		
6-1 Kyu	−1.389	0.595	<0.020	0.249	0.078–0.801
1 Dan and higher	−0.039	0.274	0.886	0.961	0.0562–1.645
Frequency of participation in competitions per year					
Do not participate^a^			0.212		
1–3 times	0.315	0.377	0.404	1.371	0.654–2.872
4–6 times	0.689	0.334	0.039	1.991	1.034–3.835
6 or more times	0.390	0.321	0.225	1.476	0.787–2.769
Gender	0.214	0.224	0.907	1.238	0.964–1.025
Age	−0.006	0.016	0.159	0.994	0.964–1.025

^a^ Dummy variable.

Thus, the model shows that the qualification of the karate athlete is significant; i.e., possessing a karate belt is important for experiencing bullying. The odds ratio (OR) of 0.249 indicates that athletes who have a 6-1 Kyu karate belt have a lower chance of experiencing bullying than athletes who have a 10-7 Kyu belt. Since the regression coefficient is negative (*b* = –1.389), there is a higher probability of experiencing bullying when the athlete has a higher qualification belt. In addition, athletes who take part in competitions 4–6 times a year have an almost two times higher chance of experiencing bullying (OR = 1.991) than those who compete only one to three times a year. Age and gender criteria do not have a significant influence on the possibility of experiencing bullying.

## Discussion

While research on the manifestation of bullying in the *Kyokushin karate* sport is lacking, this study expands knowledge in this area. According to Xu and Zhang (4), training in martial arts can reduce bullying behaviour by improving the practitioner's self-control and self-esteem, reducing hostility, and enhancing interpersonal communication abilities. However, the results of this study show that engaging in karate does not protect against bullying from other martial arts practitioners.

Three-quarters of *Kyokushin* karate athletes (75.5%) experienced at least one of the three forms of bullying, the most common being verbal bullying (42.6%). This shows that karate practitioners face a high risk of being bullied. There is a particularly high risk of experiencing verbal attacks, compared to damage in social, health and performance areas. This does not reduce the dangerousness of bullying, but may indicate certain specificity of attacks. Since we could not find similar studies in *Kyokushin* karate, the results can be compared with other studies only with caution. For example, although Kostorz and Sas-Nowosielski (51) did not study bullying, they found significantly higher physical aggression scores (hostility scores were similar) among martial arts athletes, compared to verbal aggression. Similarly, the research results of Boostani et al. (52) show that in controlling Karate (limited contact) and kickboxing (contactable) physical aggression was higher than verbal aggression. Meanwhile, hostility scores of both of them were lower than verbal and physical aggression scores. However, the authors of the above-mentioned study pointed out that behaviour in sport followed societal culture and moral norms; therefore, in the case of our study both the high indicator of bullying and the verbal bullying form that has distinguished itself may also be related to certain cultural trends. Of course, this is only an assumption that should be tested in a separate study.

The duration of practising martial arts and higher qualifications are associated with better skills and greater self-control, self-esteem, and social skills (20, 38). As discussed earlier, this should provide protection against bullying. This study also found that there were no significant differences between criteria such as possessing a belt, participating competitions, belonging to the national team, and coaching children or adults. In other words, there are no indications that there are groups with more power, such as “veterans” or “stars.” Based on Mishna et al. (53), in traditional bullying, unlike in cases of hazing, power relations are not predetermined, and the perpetrator's goal is to eliminate the victim. However, unlike in the studies by Mishna et al. (53) and Knack et al. (54), bullying was not associated with lower achievement. Novice athletes (with 10-7 *Kyu* belts) had a slightly lower chance of being bullied than athletes with 6-1 *Kyu* belts. Meanwhile, no significant differences in bullying experiences among holders of 1 *Dan* belt and above were observed at all.

*Kyokushin* karate athletes who competed more often had a greater chance of being bullied. This supports the theoretical insights in which competition is related to bullying (55, 56). Contests are associated with increased stress and strong competing. Competitive attitudes are considered one of the factors of bullying (30), and in sports where aggression is more socially acceptable, bullying behaviour may become more common (30). Notably, this study showed that athletes participating in competitions felt more socially excluded and isolated. Despite the very weak correlation between participation in competitions and social isolation, it suggests that, under conditions of increased competition, interpersonal relationships may deteriorate. This is a preliminary hypothesis that remains to be tested. Thus, it cannot be ruled out that the desire to win and lead contributed to the fact that athletes participating in competitions experienced incidents related to bullying more often.

The results of this study do not allow unambiguous confirmation of the theoretical assumptions that link martial arts to reduced bullying. Persons experiencing bullying often choose martial arts to find a way to defend themselves, reduce their anxiety, and increase their self-confidence (57). However, the results of this study demonstrate that bullying is a phenomenon that harms *Kyokushin* karate athletes. Poorer psychological state of athletes (scores of signs of stress, anxiety, and depression) was weakly correlated with damage experienced through communication and social relations in health and athletic performance. We could not find similar studies with which we could compare the statistical results, but the results of the qualitative study conducted by Dong (47) show that experienced psychological and physical violence reinforced feelings of loneliness and exclusion. Although the latter study was conducted among children, it shows, like our study, that the positive effects of karate in reducing bullying should not be overestimated, at least as far as *Kyokushin* karate is concerned.

## Conclusions

Most research to date has looked at how martial arts can contribute to tackling bullying in school-age children. The results of this study expand the age limits to include adults and fill a gap in research on bullying among *Kyokushin* karate athletes, providing new knowledge about the prevalence, forms, and effects of bullying on the emotional state. In addition, in regard to the preventive effect of martial arts on bullying and improving the emotional state, more attention must be paid to the preventing bullying between the karate athletes themselves. Because victims of bullying associate martial arts with the opportunity to defend themselves and improve their psychological state, there is a risk of entering the unsafe environment where bullying takes place. Therefore, to strengthen the preventive effect of martial arts and better protect victims, it is recommended to evaluate the experiences of persons practising martial arts not only before starting training but also during classes. In particular, *Kyokushin* coaches should pay attention to the interpersonal relations of karate athletes when preparing for competitions, which can increase the harm caused by bullying in communication, social relations, and physical health.

## Limitations of the study

This study has several limitations, which are related to the exploratory nature of the research and also highlights a few additional topics for future research. One of the limitations of this study is the small sample size and unequal respondent groups (e.g., with regard to age, duration of doing sports), which may affect the results. No quantitative studies on bullying among *Kyokushin* athletes could be found, which limited the possibility of comparing the results obtained in this study. It has not been investigated which of the persons experiencing bullying were victims before they started training; therefore, a longitudinal study is needed. Moreover, more studies such as this should be conducted, especially in culturally similar countries. This is also important because the philosophy of martial arts in Europe does not have such a religious and cultural rationale as in the countries where these sports originated.

It cannot be ruled out that cultural context could have influenced the results of this study. This study demonstrated that *Kyokushin* karate athletes encountered a high risk of bullying, especially verbal. The existence of such risk can be explained by harmful value attitudes (47, 58). Analysing how pupils reconstruct the hierarchy of masculinity in Shaolin martial arts schools, Dong (47) noted that demonstrating violence and cultivating toughness, pupils used martial masculinity models to fit in with their peers and reproduce the masculine ethos of martial arts schools. Of course, there are cultural differences between countries, but values and patterns of behaviour that are considered acceptable can be those circumstances that put karate practitioners at high risk of bullying. In any case, future examination of the relation between the values of *Kyokushin* karate athletes and violence would be useful for a better understanding of the causes of bullying in this sports branch. It would therefore make sense to apply a qualitative research design in other studies. Such a study would help answer questions such as those related to value approaches nurtured by respondents and their coaches and the ethical treatment of actions that can be classified as bullying.

## Data Availability

The raw data supporting the conclusions of this article will be made available by the authors, without undue reservation.
